# THE MEDIATING EFFECT OF BRAIN CONNECTIVITY ON DUAL-TASK PERFORMANCE: A PILOT STUDY

**DOI:** 10.1093/geroni/igae098.3746

**Published:** 2024-12-31

**Authors:** John Manning, Jennifer Yentes

**Affiliations:** Texas A&M University, Bryan, Texas, United States; Texas A&M University, Texas A&M University, Texas, United States

## Abstract

Neural connections between the prefrontal cortex and the striatum, known as cortico-striatal (CSTR) connections, are important in both cognitive tasks and gait. The purpose of this study was to investigate the mediating role of CSTR resting-state functional connectivity in the relationship between dual-tasking and gait in older adults. We made two primary hypotheses: 1) set shifting dual-task would exert a significant direct effect on gait measures and 2) task would exert a significant indirect effect on gait through CSTR functional connectivity. Five older adults completed a set shifting task where a letter-number combination (e.g. B7) was presented and subjects verbalized the number if the background was orange, or the letter if the background was blue, while standing and walking (dual-task). Later, fMRI was collected and CONN toolbox was used to calculate CSTR. A mediation analysis was performed with task as the exposure variable, CSTR as the mediator variable, and dual-task performance as the outcome variable. Results of the simple linear regression indicate no relationship between dual-task and gait (β=-0.03, p=0.62). Results of the mediation analysis indicate there is not a significant indirect effect of dual-task on gait through CSTR connectivity (i.e.=-0.024, p=0.77). This provides foundational knowledge on how these neural connections may or may not be directly related to dual-task performance during a set-shifting dual-task, which is an important step in understanding how these methods could be used for identifying early warning signs of neurodegeneration. Data collection is ongoing and updated results will be presented at the conference.

